# Association of Maternal Pre-Pregnancy Overweight and Obesity with Childhood Anthropometric Factors and Perinatal and Postnatal Outcomes: A Cross-Sectional Study

**DOI:** 10.3390/nu15153384

**Published:** 2023-07-29

**Authors:** Eleni Pavlidou, Dimitrios Papandreou, Zainab Taha, Maria Mantzorou, Stefanos Tyrovolas, Dimitrios N. Kiortsis, Evmorfia Psara, Sousana K. Papadopoulou, Marios Yfantis, Maria Spanoudaki, Georgios Antasouras, Maria Mentzelou, Constantinos Giaginis

**Affiliations:** 1 Department of Food Science and Nutrition, School of the Environment, University of the Aegean, Myrina, 81400 Lemnos, Greece; elen.p.pavl@gmail.com (E.P.); fnsd21013@fns.aegean.gr (E.P.); fnsm22031@fns.aegean.gr (M.Y.); fnsd22001@fns.aegean.gr (G.A.); maria.mentzelou@hotmail.com (M.M.); 2 Department of Health Sciences, College of Natural and Health Sciences, Zayed University, Abu Dhabi P.O. Box 144534, United Arab Emirates; dimitrios.papandreou@zu.ac.ae (D.P.); zainab.tah@zu.ac.ae (Z.T.); mantzorou.m@gmail.com (M.M.); 3 Instituto de Salud Carlos III, Centro de Investigación Biomédica en Red de Salud Mental, CIBERSAM, 28029 Madrid, Spain; stefanos.tyrovolas@polyu.edu.hk; 4 Department of Nuclear Medicine, Medical School, University of Ioannina, 45110 Ioannina, Greece; dkiorts@uoi.gr; 5 Department of Nutritional Sciences and Dietetics, School of Health Sciences, International Hellenic University, 57400 Thessaloniki, Greece; souzpapa@gmail.com (S.K.P.); maryspan1@gmail.com (M.S.)

**Keywords:** pre-pregnancy, overweight, obesity, childhood, anthropometric parameters, nutritional habits, nutritional interventions, perinatal outcomes, postnatal outcomes, healthy dietary habits

## Abstract

Background: Pre-pregnancy overweight and obesity in reproductive-aged women becomes a growing tendency in middle- and high-income populations. This study aimed to evaluate whether maternal excess body mass index (BMI) before gestation is associated with children’s anthropometric characteristics, as well as perinatal and postnatal outcomes. Methods: This was a cross-sectional study performed on 5198 children aged 2–5 years old and their paired mothers, assigned from 9 different areas of Greece. Maternal and childhood anthropometric data, as well as perinatal and postnatal outcomes, were collected from medical history records or validated questionnaires. Results: Prevalences of 24.4% and 30.6% of overweight/obesity were recorded for the enrolled children and their mothers 2–5 years postpartum. Maternal pre-pregnancy overweight/obesity was more frequently observed in older mothers and female children, and was also associated with high childbirth weight, preterm birth, high newborn ponderal index, caesarean section delivery, diabetes type 1, and childhood overweight/obesity at pre-school age. In multivariate analysis, maternal pre-pregnancy overweight/obesity was independently associated with a higher risk of childhood overweight/obesity at pre-school age, as well as with a higher increased incidence of childbirth weight, caesarean section delivery, and diabetes type 1. Conclusions: Maternal overweight/obesity rates before gestation were related with increased childhood weight status at birth and 2–5 years postpartum, highlighting the necessity of encouraging healthy lifestyle promotion, including healthier nutritional habits, and focusing on obesity population policies and nutritional interventions among women of reproductive age.

## 1. Introduction

Over recent years, the weight of pre-pregnant women, including overweight, obesity, and underweight, during childbearing ages has shown a gradually increasing trend in middle- and high-income populations [1]. The Risk Assessment Monitoring System of pregnant women, followed in different hospitals with maternity services, revealed that abnormal body mass index (BMI) before conception has increased worldwide, especially in medium- and low-income countries, including Greece. A recent cross-sectional study published in 2022 provided novel data concerning BMI incidence in terms of overweight/obesity and its determinants in 12 countries in Europe, revealing that adult obesity was the highest in Greece [2]. Obesity in Greece has been recognized as a serious public health issue; in 2015, the incidences of childhood overweight at ages 4–17 years were 22.2% and 21.6% in male and female children, respectively, while obesity was reported as 9.0% and 7.5% in male and female children, respectively [3]. Alarmingly enough, these prevalence results were amongst the greatest rates all around the world, showing a major rise (by 52%) in the incidence of childhood obesity/overweight during the previous 20 years [4].

Based on the World Health Organization (WHO), the nutritional status of mothers might be a good predictor of prolonged adverse outcomes in the women and their paired infants [5]. Notably, pre-pregnancy overweight/obesity have been recognized as crucial determinants in developing Gestational Diabetes Mellitus (GDM), hypertension, and abnormalities in fetal growth [6,7,8].

Recent research has shown that obesity has severe consequences on health because it may raise the probability of several disorders such as cardiovascular diseases, metabolic disorders, respiratory disease, mental health disturbances, and cancer [9]. Notably, mothers’ health status was directly associated with obesity, which may influence the health status of their child during the next stages of their life. Primarily, women affected by obesity before gestation may negatively influence the unborn child’s development at all stages. Several researchers investigated whether the mothers’ obesity may affect childbirth weight [10,11,12,13]. More to the point, pregnant women affected by obesity have shown a higher risk of delivering newborns of large childbirth weight (macrosomic neonate). This was mainly explained by the elevation in maternal glucose levels causing neonatal hyperglycemia and hyperinsulinemia, which accelerates fetal growth [14]. Moreover, pre-pregnancy underweight is well documented to increase the risk of premature babies and small gestational-aged newborns [15].

A systematic review demonstrated that obese pregnant women were associated with a higher risk of caesarean section and complicated births requiring instrumental delivery [16]. In addition, several research data have suggested a direct association of caesarean section incidence with obesity [17].

Moreover, mothers affected by obesity have shown some respiratory complications such as childhood asthma. This implication could be ascribed to the fact that maternal obesity might influence fetal lung development as well as the fetal immune system in the early stages of pregnancy [18]. For example, in a recent study in the UK, the researchers examined the relationship of elevated maternal BMI status with the risk of childhood health respiratory problems [19]. Similar findings were recently reported in Boston, USA, which found that obese women before gestation were associated with a higher probability of developing childhood respiratory tract infection [20].

In parallel with the association of different health problems among children born by mothers of abnormally high BMI, another study in Sweden investigated the effects of mothers’ BMI and gestational weight gain (GWG), concerning the probability of developing diabetes type 1 in children. This study indicated that women affected by obesity pre-pregnancy had a higher likelihood of diabetes type 1 occurring in their children [21]. These results have highlighted the significance of human health and antenatal treatment to promote physiological values for pregnant mothers’ BMI, to reduce the prevalence of diabetes type 1 among children. Moreover, it is reasonable that, if the parents are overweight or obese due to an unhealthy diet, there is a high probability of having overweight or obese children because their children most likely will follow similar unhealthy nutritional patterns as their family members. Accordingly, there are several substantial studies which indicate that a high proportion of women of reproductive age do not follow healthy dietary habits, such as a Mediterranean diet, in conjunction with poor lifestyle behavior (e.g., low physical activity, smoking, etc.), which may enhance the probability of carrying excess body weight [22,23,24,25].

To our knowledge, studies exploring the multifactorial impact of pre-pregnancy women with excess body weight status on childhood health complications at pre-school age in Greece still remain limited [22]. In addition, an emergently high prevalence of overweight and obesity in Greek children has been observed [3,4]. Hence, in the current cross-sectional survey, we intended to evaluate potential associations between women’s pre-pregnancy excess body weight and childhood anthropometric characteristics, as well as perinatal and postnatal outcomes. The findings of the present study derive novel evidence and data concerning pregnancy-related complications in the perinatal and postnatal period in Greek mothers and their children, which could give evidence to develop and apply new strategies, policies, and interventions to minimize this public health issue.

## 2. Methods

### 2.1. Study Population

In the current cross-sectional survey, 7047 children aged 2–5 years (pre-school stage) and their paired mothers were initially selected from 9 different areas in Greece, from kindergarten schools, playgrounds, and elsewhere. Subject data collection was performed between May 2016 and September 2020. The principles for the primary assignments included children aged 2–5 years. Moreover, their mothers should have a singleton childbirth prior to assignments, independently of parity. The enrolled women had no other gestation during the period of time from this singleton childbirth to the time of study, i.e., 2–5 years after delivery. Both children and their mothers were disease-free during the postpartum period.

Seven thousand forty-seven (7047) children and their paired mothers were initially enrolled in this study. Nine hundred eighty-eight (988) of them (14.0%) were missing or had incomplete data and were, thus, excluded from the study. Sample size estimation was utilized by applying PS: Power and Sample Size estimator software. A simple randomization method was applied using a sequence of random binary numbers (e.g., 100, 101, 011, in which 0 was characterized as assignment and 1 non-assignment to this study).

Among the remaining 6059 subjects, 861 (14.2%) children were afterwards not included because of a previous other disorder/pathological condition such as hyperinsulinemia, low hematocrit, high lipid levels, type 2 diabetes, cancer, autism spectrum disorder, motor disorder, or mental retardation. The above elimination criteria were used to determine if pre-pregnancy women overweight/obesity may independently influence the incidence of children overweight/obesity, since other children’s disorders could affect the probability of overweight/obesity in children, such as cardiometabolic diseases, for which a greatly elevated study population should be recommended. In addition, the mothers of the enrolled children did not have any disease during their pregnancy, except for any history of gestational diabetes or gestational hypertension which were cured efficiently during their gestation.

Neonatal ponderal index was also measured as per protocol [23]. Mothers reported whether they exhibited a preterm birth (<37th week) and the data were cross-tested with their medical records. However, we found that there were some lost data concerning the exact week of preterm delivery in their medical files and some data did not agree with the women’s answers; therefore, preterm birth was treated as a binary variable, i.e., either prior to or after the 37th week of gestation.

Finally, 5198 children and their paired mothers were enrolled in the study, leading to a 73.8% final response rate. We provided detailed information to all participants concerning the scope of the study, and all participating mothers accepted to sign a written informed consent. The study was in compliance with the World Health Organization (52nd WMA General Assembly, Edinburgh, Scotland, 2000) and approved by the Ethical Organization of the University of the Aegean (ethical approval code: no 12/14.5.2016).

### 2.2. Study Design

Certified semi-quantitative questionnaires were applied for collecting the anthropometric and demographic data as well as the perinatal and postnatal outcomes of the enrolled individuals [23,24,25] 2.5 years postpartum. Data on childhood anthropometry such as childbirth weight, height, and head circumference were retrieved by women’s gynecologists’ or hospitals’ medical records. Children’s birth weight of <2500 g was classified as low, 2500–4000 g as normal, and >4000 g as high. Trained dietitians and nutritionists collected all childhood anthropometry data (weight and height) 2–5 years postpartum, as per protocol [24,26,27]. Mother’s and children’s weight was determined utilizing a Seca scale [Seca, Hanover, MD], without shoes, to the nearest 100 g, and height was determined utilizing a portable stadiometer (GIMA Stadiometer 27335) with no shoes on, to the nearest 0.1 cm. Two international datasets were applied for defining overweight and obesity in both children and their paired mothers: the WHO and the International Obesity Task Force (IOTF) data recommendations [28,29]. Τhe kind of delivery (vaginal or caesarean section), childhood type 1 diabetes mellitus, and asthma data were also recorded from the given questionnaire. The childhood asthma data were reported by a certified pediatrician physician, according to the International Study of Asthma and Allergies in Children guidelines’ recommendations [30].

Mothers’ anthropometric data during the first weeks of gestation were measured during a visit to their individual gynecologists or to public or private hospitals. Both weight and height data for women during the first weeks of gestation were extracted from their medical files; thus, they had been measured and were not being self-reported. Mothers’ pre-pregnancy BMI was estimated according to the weight and height measured during the first weeks of pregnancy. Detailed information was provided to all participating mothers by trained dietitians and nutritionists concerning the accomplishment of questionnaires by a face-to-face interview. The trained personnel provided a comprehensive description of the questions, to increase the accuracy of responses.

### 2.3. Statistical Analysis

Student’s *t*-test was applied for continuous variables, which were normally distributed. Normality of distribution was evaluated by Kolmogorov–Smirnov test. Categorical variables were assessed by Chi-square. The quantitative variables following normal distribution were given as mean value ± Standard Deviation (SD). The quantitative continuous variables, which did not follow a normal distribution, are provided as median value (Interquartile Range, IQR). The qualitative variables are stated as absolute or relative incidences. To determine if maternal pre-pregnancy BMI status, as the main independent variable, was independently associated with multiple dependent variables, including children anthropometry data and childhood perinatal and postnatal outcomes, multivariate logistic regression analysis was conducted by adjusting for possible confounders such as maternal age; children’s gender, birth weight, height, and head circumference; preterm birth; newborn ponderal index; type of delivery; and childhood diabetes type 1, which showed significant effects previously in pre-pregnancy maternal BMI in univariate analyses. The Statistica 10.0 software was used for performing the statistical analysis of the survey data (Informer Technologies, Inc., Hamburg, Germany).

## 3. Results

### 3.1. Anthropometry Data and Perinatal and Postnatal Outcomes of the Study Population

**Figure 1:**
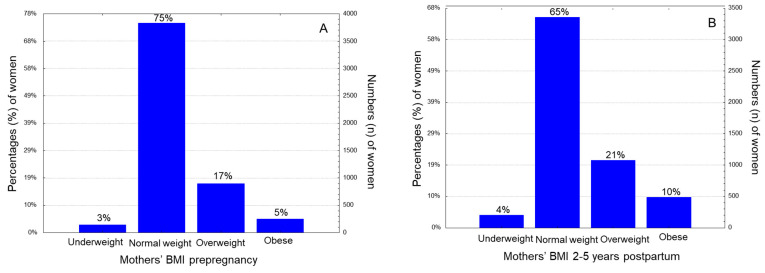
Histogram distributions of women under study according to (**A**) Mothers’ BMI before gestation and (**B**) Mothers’ BMI “2–5” years postpartum.

The current cross-sectional survey included 5198 children aged 2–5 years and their paired mothers, who were selected 2–5 years postpartum. A total of 17.4% of the mothers were overweight and 4.9% were affected by obesity before pregnancy, based on their BMI classification (Figure 1A). At the time of study, 2–5 years after delivery, 21.0% of them were overweight and 9.6% were obese (Figure 1B). Overall, 22.3% of participating women were overweight/obese pre-pregnancy and this prevalence was significantly increased to 30.1% 2–5 years postpartum (*p* < 0.0001). Regarding the type of delivery, 43.7% delivered vaginally and 56.3% by caesarean section. Preterm birth was noted in 30.0% of the women under study. Newborn ponderal index was classified as high in 47.8% of the women under study.

**Figure 2:**
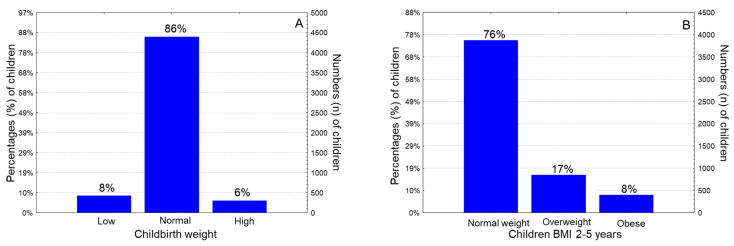
Histogram distribution of children under study according to (**A**) Childbirth weight and (**B**) Children’s BMI “2–5” years postpartum.

The mean childbirth weight was 3152 ± 461 g (range: 1320–5000 g), the mean childbirth height was 46.4 ± 2.7 cm (range: 42–52 cm), and the mean childbirth head circumference was 36.0 ± 2.0 cm (range: 32–39 cm). Grouping the children based on childbirth weight, 8.3% were classified as low newborn weight (<2500 g), 85.7% exhibited normal newborn weight (2500–4000 g) and 6.0% exhibited high newborn weight (>4000 g) (Figure 2A). Regarding children’s BMI at 2–5 years old, 16.5% of them were affected by overweight and 7.9% were affected by obesity; overall, 24.3% of the children were overweight/obese (Figure 2B).

The mean children’s age was 4.1 ± 1.2 years (range: 2.0–5.5 years). Regarding children’s gender, 49.3% of them were male and the rest (50.7%) were female. The mean childbirth weight was 3152 ± 461 g (range: 1320–5000 g), the mean childbirth height was 46.4 ± 2.7 cm (range: 42–52 cm), and the mean childbirth head circumference was 36.0 ± 2.0 cm (range: 32–39 cm). Based on their birth weight, 8.3% of children were classified as low newborn weight (<2500 g), 85.7% of children exhibited normal newborn weight (2500–4000 g), and 6.0% of children exhibited high newborn weight (>4000 g) (Figure 2A).

Postnatal outcomes revealed that 4.3% and 4.5% of the children were diagnosed with diabetes type 1 and asthma, respectively. Regarding children’s BMI at 2–5 years old, 16.5% of them were classified as overweight and 7.9% of children were affected by obesity; as such, 24.4% of them were affected by overweight or obesity overall (Figure 2B).

### 3.2. Maternal Pre-Pregnancy Excess Weight in Association with Antrhopometric and Demographic Characteristics of the Participant Children

Childhood obesity at 2–5 years old was significantly more frequently observed when maternal BMI before gestation was overweight or obese (Table 1, *p* < 0.0001). Childbirth weight was significantly increased in those children whose mothers were affected by overweight or obesity before gestation than newborns whose mothers were underweight or normal weight before gestation (Table 1, 3269 ± 483 g vs. 3107 ± 412 g, *p* = 0.0172). Newborns with high weight status were significantly more frequently observed in children whose mothers were obese before gestation (Table 1, *p* = 0.0001). Mothers’ BMI status before gestation did not show any relationship with childbirth height or head circumference (Table 1, *p* > 0.05). Mothers affected by overweight or obesity before gestation were significantly older than underweight and normal weight mothers (Table 1, *p* = 0.0001). Preterm birth was noted in 30.0% of the women under study.

### 3.3. Maternal BMI Status before Gestation in Relation with Childhood Perinatal and Postnatal Outcomes

Children whose mothers had an abnormal pre-pregnancy BMI (meaning they were overweight or obese) were significantly more frequently delivered by caesarean section compared to those whose mothers were underweight or normal weight before gestation (Table 1, *p* = <0.0001). Moreover, children whose mothers were affected by overweight or obesity before gestation showed a substantially greater incidence of being diagnosed with diabetes type 1 (Table 1, *p* = 0.0009). Mothers’ BMI status before gestation was not associated with the prevalence of children to develop asthma at the ages 2–5 years (Table 1, *p* > 0.05). Moreover, we did not find any significance difference between childbirth weight status and childhood asthma at the ages of 2–5 years (*p* > 0.05, data not shown).

### 3.4. Pre-Pregnancy BMI Status-Multivariate Regression Analysis

Multivariate logistic regression analysis revealed that maternal overweight and obesity before gestation was significantly independently related with childhood BMI at 2–5 years old, childbirth weight status, type of delivery, and childhood diabetes type 1 (Table 2, *p* = 0.0001, *p* = 0.0009, *p* = 0.0175, and *p* = 0.0014, respectively). Mothers affected by overweight or obesity prior to gestation exhibited more than 2-fold higher odds for delivering children who were affected by overweight or obesity at 2–5 years old (Table 2, *p* = 0.0001). Moreover, mothers affected by overweight or obesity before pregnancy presented a 95% higher probability of delivering children with high newborn weight (Table 2, *p* = 0.0009).

Mothers affected by overweight or obesity before pregnancy also exhibited a 71% higher likelihood of delivering their children by caesarean section (Table 2, *p* = 0.0175). Furthermore, mothers affected by overweight or obesity before pregnancy had a 28% higher risk of delivering children who developed diabetes type 1 over the next 2–5 years of their life (Table 2, *p* = 0.0014).

Maternal age, childhood gender, preterm birth, and newborn ponderal index did not remain significant in multivariate analysis (Table 2, *p* > 0.05).

## 4. Discussion

The present study reported that maternal overweight and obesity before gestation were related, with high rates of overweight and obesity in their offspring aged 2–5 years. Our data also showed that children whose mothers were affected by overweight or obesity before gestation exhibited higher incidence of high birth weight. In addition, a higher incidence of delivery by caesarean section was recorded when the mothers were affected by overweight or obesity before gestation. Moreover, children whose mothers were affected by overweight or obesity before gestation exhibited higher propensity to developing diabetes type 1 in their later life. All the above associations remained significant in multivariate analysis, after adjusting for multiple confounding factors. These results highlight the importance of early prevention of obesity among Greek reproductive-aged women, to limit adverse health outcomes linked to their children at short- and long-term levels such as excess birth weight status, childhood obesity, and diabetes type 1.

Alarmingly enough, our data supported evidence that children 2–5 years old were more likely to be affected by overweight or obesity when their mothers had overweight or obese BMI status pre-pregnancy. This observation is in line with previous research in this field [31] among children of a similar age. Previous substantial studies have further supported that maternal obesity during the pre-pregnancy period, during pregnancy, and postpartum may be related with higher risk of obesity rates among their children [32]. In this aspect, literature data showed that maternal pre-pregnancy obesity may also be related with increased total and visceral fat among children 9–13 years old [33]. In line with the above findings, our analysis showed that high newborn weight (>4000 g) was more frequently observed in children whose mothers were affected by overweight or obesity before gestation. In support of the previous findings, the bio-physiological pathway of insulin resistance among overweight and obese mothers may lead, among other factors, to elevated glucose levels during gestation, which was linked with accelerated fetus development and large birth weight for newborns [34]. Further data have also supported the pathway of in uterus child obesity “programing”, where the mother’s obesity and abnormal metabolic status may play an important role [35]. The above findings, in conjunction with the high rates of obesity in Greece among children and among women of childbearing age, may raise a red flag concerning the need for early obesity prevention public programs and policies at early stages before gestation, to battle the intergenerational cycle of excess body weight and abnormal metabolic outcomes.

Furthermore, the positive association of the excess weight (overweight and obesity) at pre-pregnancy timing with childhood anthropometric and demographic characteristics, as well as perinatal and postnatal outcomes, was assessed by adjusting for several potential confounding factors. When the multivariable modelling was applied, maternal overweight and obesity before gestation was related with a high probability of childhood overweight/obesity at 2–5 years old, high birth weight status, caesarean type of delivery, and childhood diabetes type 1. More specifically, the analysis showed that women affected by overweight or obesity prior to gestation exhibited more than a 2-fold higher risk of delivering children who were affected by overweight or obesity at 2–5 years old. Our analysis is in accordance with former surveys supporting the finding of a pre-pregnancy association with child obesity [32,36]. Among our findings, pre-pregnancy obesity was related with caesarean delivery type. Previous meta-analyses and observational studies also support our finding concerning the relationship between maternal obesity before pregnancy and the rates of caesarean sections [37,38]. However, caesarean section usually is strongly necessitated for medical reasons, especially when there is a high probability for severe complications compared to vaginal delivery, such as extreme maternal obesity, preeclampsia, gestational diabetes or hypertension, or chronic diseases such as thyroid gland disorders, cardiovascular disease, cancer, multiple sclerosis, or other autoimmune diseases [36,37,38].

Moreover, our data analysis showed that women affected by overweight or obesity before pregnancy exhibited a 28% higher probability of delivering children who developed diabetes type 1 during the next stages of their life. The above result is in accordance with several surveys; however, inconsistent findings have been reported so far [39], which strongly emphasizes the necessity for additional research into the underlying mechanisms. Identification of pre-, peri-, and post-natal factors and their influence on maternal and offspring health is of great importance, as there is a gap in the international literature concerning this issue, especially in Greece. Such evidence may contribute to design and application of future inter-generational prevention and management programs for mother–child health in the Greek population, as well as in the whole Mediterranean region.

Recent studies mark the beneficial role of nutrition and physical activity interventions in obesity and metabolic abnormalities among mothers and their offspring [40,41]. The health and medical community in Greece should recognize excess weight as among the priority mutual risk factors for the country’s reproductive-aged women and their newborn children. Targeted physical activity and nutritional recommendations according to the Mediterranean diet (that is, the healthier nutritional patterns of the region) for women during the pre-pregnancy period, as well as during pregnancy and at the post-pregnancy stage, could serve as cost-effective planning for maternal (and their offspring’s) disease prevention [23,24,27].

There is currently strong evidence concerning the need to design and promote forthcoming efficient strategies and approaches to limit early obesity. This constitutes a strong demand, which may improve lifestyle behaviors such as infant eating behaviors, contributing also to define the potential impact of other mechanisms, such as the impact of perinatal factors including mothers’ nutritional habits during gestation, epigenetics, or microbiome [42]. Interestingly, a recent meta-analysis showed that Mediterranean diet-based interventions may exert a considerable impact on lowering the BMI and decreasing obesity in children and adolescents at ages between 3 and 18 years old [43].

To date, nutritional monitoring and support have been performed in several interventional surveys related with obesity during childhood. Various methods of nutritional training and advising, key messages, Mediterranean-style nutritional patterns with fewer calories, and healthier food choices have been applied as nutritional interventions [44]. However, the currently available data for Greek children remain extremely scarce. In this aspect, it is in our plans to perform similar studies in children and adolescents in our country—Greece. The Mediterranean diet includes daily intake of whole cereals, fruit, vegetables, and legumes in specified amounts, weekly consumption of white meat in low amounts, and, occasionally, sweets and chocolates in small amounts. Notably, extra virgin olive oil, which constitutes the major lipids source in a Mediterranean diet, has shown beneficial healthy effects for human health. Moreover, diverse fruits and vegetables, which include high amounts of phytochemicals, constitute a main proportion of this dietary pattern, contributing to the total nutritional value and bioactivity of its components [45,46].

It is reasonable to expect that our study had certain limitations, such as the recall bias of the mothers when reporting the data, especially for self-reported questions. Moreover, conclusive data regarding causality cannot be provided, due to the cross-sectional design of the current survey, despite its representative and large study population. Another limitation lies with BMI measurement, as it is considered as a weak tool to calculate body fat because it cannot directly assess, especially, its body distribution. Thus, direct measures of body fat mass using bioelectrical impedance analysis or skinfold measurements will be beneficial in future studies to confirm our findings. The lack of physical activity data and mental status information could also affect our results. Nevertheless, our study is one of the few reported in Greece that has attempted to evaluate pre-pregnancy overweight and obesity and their effect on childhood demographic and anthropometric characteristics, as well as on perinatal and postnatal outcomes in a large representative sample of the Greek population of pre-school children. Moreover, in our study, we explored if mothers’ obesity may affect the incidence of obesity in their paired children, independently of other childhood diseases, which could elevate the probability of developing obesity in children, along with metabolic diseases, for which a significantly higher sample size is obligatory. In this context, it should be noted that future studies should be conducted on a more appropriate sample size to address the impacts of such diseases in childhood obesity.

## 5. Conclusions

Maternal pre-pregnancy overweight and obesity rates have been related to elevated children’s BMI in pre-school ages (i.e., 2–5 years old), birth weight status, caesarean type of delivery, and childhood diabetes type 1. These results highlight the significance of developing novel approaches for preventing obesity among mothers at pre-pregnancy stages by decreasing various adverse perinatal maternal risk factors, as well as by minimizing their offspring’s adverse health-related outcomes. Considering that obesity among young women and children is recognized as a major global wellbeing challenge, particularly in Greece and Mediterranean countries, the above results emphasize the crucial requirement for a change towards a healthier lifestyle, including healthier nutritional habits. Additionally, public health policies and interventions should be targeted towards the obese population among women of reproductive age, to optimize a metabolic healthy inter-generational lifecycle. Moreover, it should be noted that obesity in children enhances the probability of developing several disorders such as diabetes mellitus, cardiovascular diseases, endocrine disorders, autoimmune disorders, cancer, etc., at the next stages of children’s lives. Thus, public health strategies should inform their mothers, in order to reduce the childhood obesity pandemic. Public policies are strongly recommended to inform reproductive-aged women as to the negative effects of childhood obesity concerning the next stages of their lives.

## Figures and Tables

**Table 1:** Associations between maternal BMI status before gestation with childhood anthropometry data and perinatal and postnatal outcomes.

Characteristics (*n* = 5198)	Pre-Pregnancy BMI Status *		
	Underweight and Normal Weight (69.4%)	Overweight and Obese (30.6%)	*p*-Value
Maternal Age (years ± SD)	33.1 ± 1.12	36.8 ± 1.21	*p* = 0.0001
Offspring Gender (*n*, %)			*p* = 0.0001
Male	2053 (50.9)	510 (43.7)	
Female	1979 (49.1)	656 (56.3)	
Childhood BMI categories at the age of 2–5 years (*n*, %)			*p* < 0.0001
Normal weight	3103 (77.0)	826 (70.8)	
Overweight	675 (16.7)	184 (15.8)	
Obese	254 (6.3)	153 (13.4)	
Birth weight (g)	3107 ± 412	3269 ± 483	*p* = 0.0172
Birth weight status *(n*, %)			*p* = 0.0001
Low newborn weight (<2500 g)	341 (8.5)	92 (7.9)	
Normal newborn weight (2500–4000 g)	3480 (86.3)	972 (83.4)	
High newborn weight (>4000 g)	211 (5.2)	102 (8.7)	
Birth height (cm)	46.4 ± 2.9	46.5 ± 1.9	*p* = 0.8457
Birth head circumference (cm)	36.0 ± 2.1	36.1 ± 1.6	*p* = 0.5107
Preterm childbirth (<37th week, *n*, %)			*p* < 0.0001
No	1920 (84.9)	1701 (58.3)	
Yes	342 (15.1)	1219 (41.7)	
Newborn ponderal index (*n*, %)			*p* < 0.0001
Low	2327 (57.7)	485 (41.6)	
High	1805 (42.3)	681 (58.4)	
Type of delivery (*n*, %)			*p* < 0.0001
Vaginal	1845 (45.8)	426 (36.5)	
Caesarean section	2187 (54.2)	740 (63.5)	
Childhood asthma (*n*, %)			0.5292
No	3845 (95.4)	1117 (95.8)	
Yes	187 (4.6)	49 (4.2)	
Childhood diabetes type 1 (*n*, %)			*p* = 0.0009
No	3881 (96.3)	1096 (94.0)	
Yes	151 (3.7)	70 (6.0)	

* Chi-square test was applied for examining categorical variables, t-student test for normally distributed continuous variables between two groups, ANOVA for normally distributed continuous variables between three groups.

**Table 2:** Multivariate logistic regression analysis model of mothers’ overweight and obesity before gestation and childhood anthropometry data, after adjusting for possible confounding factors.

Characteristics	Pre-Pregnancy Overweight and Obesity	
	OR * (95% CI **)	*p*-Value
Maternal age (Under/Above mean value)	1.62 (0.89–2.12)	*p* = 0.0884
Childhood Gender (Boys/Girls)	1.07 (0.32–1.88)	*p* = 0.3029
Childhood BMI at the age of 2–5 years (Normal/Overweight or obese)	2.11 (1.80–2.42)	*p* = 0.0001
Childbirth weight status (Low & normal/High newborn weight)	1.95 (1.61–2.53)	*p* = 0.0009
Preterm childbirth (<37th week, No/Yes)	1.34 (0.58–2.34)	*p* = 0.4378
Newborn ponderal index (Low/High)	1.47 (0.69–2.10)	*p* = 0.5745
Type of delivery (Vaginal/Caesarean section)	1.71 (1.33–2.19)	*p* = 0.0175
Diabetes type 1 (No/Yes)	1.27 (1.04–1.53)	*p* = 0.0014

* OR: Odds Ratio; ** CI: Confidence Interval.

## Data Availability

Available upon request to the corresponding author.
